# A case of mesh plug migration into the bladder 5 years after hernia repair

**DOI:** 10.1186/s40792-014-0004-2

**Published:** 2015-01-16

**Authors:** Shinji Ishikawa, Takashi Kawano, Ryuichi Karashima, Tetsumasa Arita, Yasushi Yagi, Masahiko Hirota

**Affiliations:** Department of Surgery, Kumamoto Regional Medical Center, 5-16-10 Honjo, Kumamoto City, Kumamoto 860-0811 Japan; Kawano Hospital, 6-25-1 Ooe, Kumamoto City, Kumamoto 862-0971 Japan; Department of Gastroenterological Surgery, Graduate School of Medical Sciences, Kumamoto University, 1-1-1 Honjo, Kumamoto, 860-8556 Japan

**Keywords:** Mesh plug, Inguinal hernia, Bladder, Migration

## Abstract

The mesh plug technique is one of the most popular procedures for inguinal hernia repair in Japan. This procedure is quick, easy, and low cost with a low recurrence rate. However, some complications associated with the mesh plug have been reported recently. We hereby present a case of an 80-year-old female admitted to our hospital with swelling and pain in the right lower abdomen 5 years after hernia repair with the PerFix plug. Discharge of urine through the route of exploratory needle puncture demonstrated the fistula of skin and the bladder. Computed tomography (CT), magnetic resonance imaging (MRI), abdominal US, and cystoscope examination revealed that the mesh plug had penetrated into the bladder. As far as we know, this is the first report that a mesh plug has migrated into the bladder.

## Background

Endoscopic repair or Lichtenstein method is now recommended for inguinal hernia repair by the European Hernia Society Guidelines [1]. However, hernia repair using a mesh plug is more common in Japan, and more than 80% of adult inguinal hernia cases have been treated by using this technique in our hospital. The reasons are being a quick procedure, easy to learn, and also low at cost. Recurrence (due to the shrink of the mesh) and prolonged pain [2] have been reported as the main complications. However, some rare complications have emerged recently. There were five reported cases that indicated the complication being caused by mesh plug migration.

In one case, the plug had migrated away from the left internal ring in the preperitoneal space 18 months after inguinal hernia repair [3]. Three cases showed perforations or fistulas into the intestinal tract [4-6]. The fifth case report described a migration into the scrotum [7]. However, there was no report similar to our case.

We herein report a complicated case of mesh plug migration into the bladder 5 years after hernia repair with PerFix Plug (Bard Co., Murray Hill, NJ, USA).

## Case presentation

An 80-year-old female was admitted to our hospital with right inguinal pain and fever. She underwent a direct inguinal hernia repair with PerFix Mesh 5 years before. Swelling around the operation scar was observed. An infection of the mesh was suspected so an exploratory needle puncture was performed. Infected discharge was observed at first, but 30 min later, urine was discharged from the punctured hole. A bladder skin fistula was suspected, and careful examination was done. Unusual findings were not observed except for a slight elevation of WBC (11,200/mm^3^) and CRP (3.3 mg/dl) by laboratory profile.

**Figure 1:**
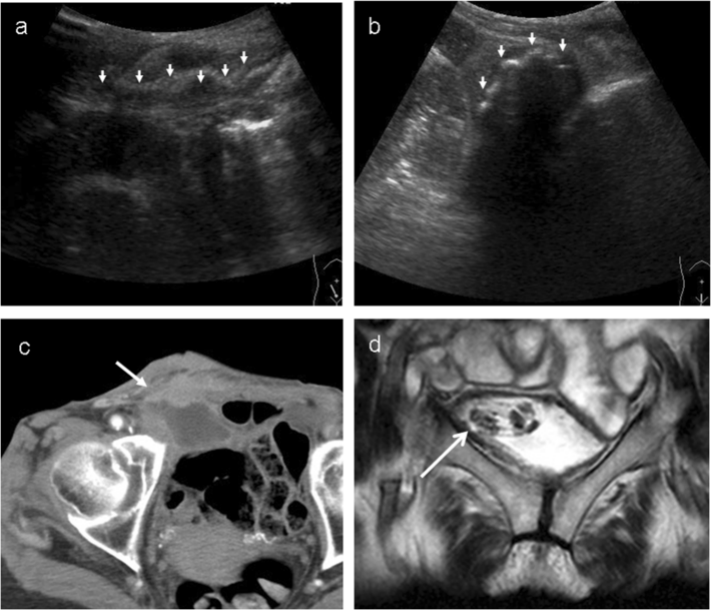
**Image findings of mesh plug penetrated into the bladder.** Abdominal ultrasound tomography detected **(a)** onlay mesh and **(b)** mesh plug. **(c)** Computer tomography showed high-density area (mesh plug) in the subcutaneous of right inguinal lesion. **(d)** Magnetic resonance imaging detected the mesh plug penetrated into the bladder.

**Figure 2:**
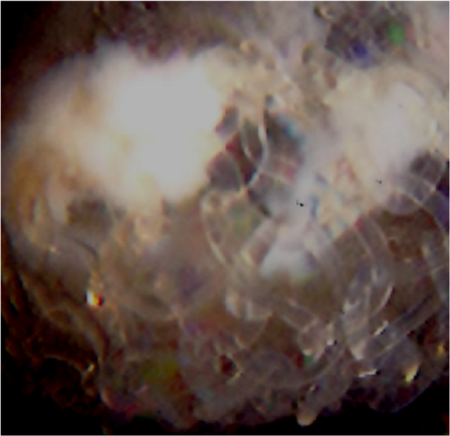
**The cystoscope revealed that the mesh plug penetrated into the bladder.** The entire image shows the penetrated mesh plug. Surrounding is the bladder lumen.

Abdominal ultrasound tomography located both the onlay and mesh plug and revealed a fluid collection surrounding the onlay mesh (Figure 1a,b). Computer tomography (CT) showed a high-density area in the subcutaneous area of the right inguinal lesion (Figure 1c). Magnetic resonance imaging (MRI) revealed that the mesh plug had penetrated into the bladder (Figure 1d). The cystoscope study confirmed the MRI findings (Figure 2). We diagnosed the condition as a penetration of the mesh plug into the bladder and mesh infection.

**Figure 3:**
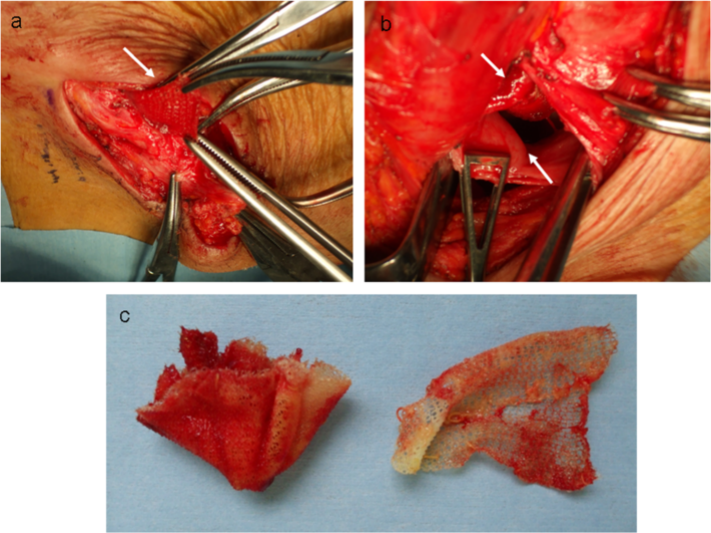
**Operative findings. (a)** The removal of onlay mesh (arrow). **(b)** The arrow head above shows the mesh plug. The arrow head below shows the wall of the bladder. **(c)** Removed mesh plug (right) and the onlay mesh (left).

Removal of the mesh and partial resection of the bladder was performed. The fistula route and the granulation around the mesh were also resected. Though the onlay mesh was removed from the inguinal incision (Figure 3a), the mesh plug was not visible. We performed an additional laparotomy in the mid lower abdomen and removed the mesh plug from the bladder (Figure 3b) and repaired the bladder. No complication after surgery was observed. Any recurrence of hernia has not been observed by now.

## Discussion

Recently, there are several reports concerning complications after hernia repair with a mesh plug. According to the reports, mesh migration was observed in the preperitoneal space, small intestine and colon, and scrotum [2-7]. There was one case that reported that the mesh had migrated into the bladder after endoscopic repair [8] but we could not find any reports that the mesh plug migrated into the bladder by searching English language MEDLINE. In our case, more than half of the mesh plug had penetrated into the bladder, pointing to a mesh plug migration into the bladder.

The question is how mesh plug migration occurs. In a review article on mesh plug migration cases, the authors claim that surgical technique was poor in some cases, one case did not show true migration, another one was a case of the wrong operation being done, and one case due to the patient's very poor health [9]. They concluded that this kind of complication could be avoided by careful operation. There are no obvious reasons why the mesh plug migrated into the bladder in our case. However, when considering the reported reasons above, it might be possible to make a hypothesis. First, the body weight of this patient was 37 kg at the time of the inguinal hernia operation. As she also suffered the aftereffects of a subarachnoidal hemorrhage, it can be assumed that the patient's health was poor. This condition may cause problems to sufficiently fix the mesh as other reports indicated [7,9]. Second, as the inguinal hernia was a direct hernia, the mesh plug was inserted into the transversal fascia, and this position is in the vicinity of the bladder. Due to the patient's poor health, it is easy to imagine that the adipose tissue between the mesh and the bladder might have been thin, and this caused a condition in which the mesh plug was pressed against the bladder wall each time the urine was collected in the bladder. This might have ruined the fixation of the mesh plug and finally the hard tip of the mesh plug penetrated the bladder and migrated.

Furthermore, we found that the removed mesh plug and the onlay mesh slightly shrunk compared to their original size but not stiffened as reported [2] and with no granulation attracted (Figure 3c). This suggests that the penetration into the bladder might have occurred far earlier, and the wet environment prevented the mesh from becoming stiffened and from developing granulation. Perhaps, a genitourinary infection has occurred at a later point of time, and the mesh was then infected.

We changed the type of the mesh plug to ProLoop plug (Atrium, Hudson, NH, USA) after we experienced this case, a far less stiff mesh plug than the formerly used PerFix plug. Though there is not yet sufficient report on that, we have come to believe that this type of complication is at least partially caused by the stiffness of the mesh plug. At least in cases like ours, a soft plug should ease the tension between the bladder and the fixation of the plug compared to a hard type plug. This might prevent damage to the fixation site and also prevent the penetration into the bladder and, thus, may avoid this complication altogether. We are fully aware that open methods such as Kugel or Prolene hernia system might prevent this kind of complication in the first place. However, the mesh plug system is quick, easy to learn, and low cost, so we consider this one of the standard procedures for inguinal hernia repair. Furthermore, a randomized control study comparing Lichtenstein patch, PerFix plug, and ProLoop plug [10] reported that though there were no significant differences among the groups in operative time, hospital stay, bodily pain scores, and complication rates, the authors concluded that the ProLoop plug is a safe and effective method of repairing inguinal hernias. Its novel lightweight configuration does not increase the risk of recurrence when compared to thicker mesh plugs, and it may offer benefits in patient comfort.

## Conclusions

As far as we know, this is the first report that the mesh plug migrated into the bladder. Though this type of complication is rare, there are a great number of patients that received an operation by this procedure; we must keep in mind that this type of complication might occur.

## Consent

We confirmed that the patient's next-of-kin agrees with publishing this case as a case report.
